# Spindle cell sarcomatoid carcinoma of the trachea: first case report of surgical resection

**DOI:** 10.1186/s13019-016-0524-x

**Published:** 2016-08-05

**Authors:** Juan P. Gurria, David M. De Acosta, Niloufar Hafezi, Eman B. Yousif, Ehab AlAmeer, Richard C. Anderson

**Affiliations:** Department of Surgery, University of Illinois College of Medicine at Peoria, 624 N.E. Glen Oak Avenue, Ste. 2680, Peoria, IL 61603-3135 USA

**Keywords:** Spindle cell, Sarcomatoid, Carcinoma, Trachea, Tumor, Resection

## Abstract

**Background:**

Primary malignant tracheal tumors are rare, accounting for approximately 0.2 % of respiratory tract tumors yearly, with squamous cell carcinomas and adenoid cystic carcinomas accounting for two-thirds of these cases. Sarcomatoid carcinomas are a group of poorly differentiated non-small cell lung carcinomas containing a component of sarcoma or sarcoma-like (spindle and/or giant cell) differentiation, categorized into five morphologic subgroups. Spindle cell sarcomatoid carcinoma is a rare variant of sarcomatoid carcinomas, consisting of only spindle-shaped tumor cells. Only one other case has been reported as a primary tracheal tumor.

**Case presentation:**

We present a 75-year-old male, having progressive dyspnea and cough, with a spindle cell sarcomatoid carcinoma tumor visualized on chest computed tomography scan and confirmed with biopsy.

**Conclusions:**

Due to its low incidence, knowledge of treatment methods, prognostic factors, and etiology is limited thus approaches to eradication have widely varied. We are reporting the second published case of spindle cell sarcomatoid carcinoma of the trachea and the first reported successful outcome of definitive treatment with tracheal resection.

## Background

Primary tracheal tumors are rare, with malignancy occurring at a rate of 0.1 new cases per 100,000 every year, corresponding to 0.2 % of all tumors involving the respiratory tract [1]. Primary tracheal cancers are more common in current or former male cigarette smokers, with peak incidence in the sixth decade of life [2, 3]. Due to the central location and tendency to protrude into the lumen of the large airways, patients typically present with symptoms of dry cough, hemoptysis, progressive dyspnea, hoarseness, and fevers secondary to recurrent pneumonia, often leading to misdiagnosis of asthma or chronic pulmonary disease [4, 5]. Squamous cell carcinoma (SCC) and adenoid cystic carcinoma (ACC) make up nearly two thirds of primary tracheal cases. Tracheal tumors apart from these are extremely uncommon and consist of a heterogeneous group of benign and malignant tumors, with only one other reported case of primary tracheal spindle cell carcinomas to date [6, 7].

Spindle cell carcinoma belongs to a group of poorly differentiated non-small cell carcinomas known as sarcomatoid carcinomas. According to the World Health Organization 2004 classifications of lung tumors, this group contains components of sarcoma or sarcoma-like (spindle cell or giant cell) differentiation, consisting of only spindle shaped cells that form cohesive nests and irregular fascicles of malignant cells with features including nuclear hyperchromasia and distinct nucleoli [4]. These tumor masses include scattered and focally dense regions of lymphoplasmacytic infiltrates throughout [4].

Due to the rarity of these tracheal cases, there is often a delay in diagnosis and inappropriate treatment approaches [2]. Additionally, no universally accepted TNM classification for tracheal malignancies exists, and proposed staging systems are based on a small number of considered cases [1, 8]. Generally, tracheal carcinomas have poor prognosis, with 5-year survival rates of up to 15 % and 10-year survival rates of 7 % [1]. On the other hand, the spindle cell group of carcinomas are described to be aggressive and thought to have worse clinical outcomes than conventional non-small cell carcinomas. Five-year survival rates are only 20 % despite many patients presenting with stage I disease [4]. Adjuvant chemotherapy and radiation do not seem to play a helpful role in treatment of spindle cell type tumors, and thus resection remains the mainstay of tumor eradication [4]. Here we report a case of a former smoker presenting with progressive dyspnea, discovered to have primary tracheal spindle cell sarcomatoid carcinoma treated definitively with surgical resection.

## Case presentation

A 75 year-old Caucasian male presented with progressively worsening dyspnea of eight days, wheezing and productive cough with clear sputum. His symptoms began abruptly, worsened upon exertion, and were unrelieved by home albuterol inhaler. The patient is a former smoker with a 52 pack-year history, quitting four years prior to the onset of his symptoms. His past medical history is significant for chronic obstructive pulmonary disease (COPD) and atherosclerotic disease involving his peripheral and carotid arteries. His past surgical history is unremarkable, and lungs were clear to auscultation with stridor on physical exam at the time of his presentation.

**Fig. 1:**
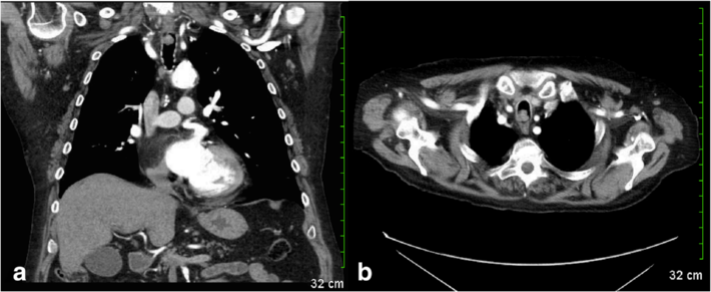
**a** (left): Chest computed tomography scan sagittal view demonstrating a 1.5 cm nodular soft tissue lesion within the upper subglottic thoracic trachea, 8 cm from carina. **b** (right): Axial view of the lesion resulting in greater than fifty-percent reduction in cross-sectional area of the tracheal lumen

A chest computed tomography (CT) scan was performed with contrast demonstrating a 1.5 cm x × 1.3 cm × 1.3 cm nodular soft tissue lesion within the subglottic trachea, causing 50 % reduction in cross-sectional area of the tracheal lumen (Fig. 1). The patient subsequently underwent bronchoscopy. A biopsy of the tracheal mass was simultaneously obtained, with a preoperative diagnosis of T1aN0M0 primary spindle cell sarcomatoid-type carcinoma [9]. The patient’s lesion involved trachea of resectable length, without ingrowth to irresectable structures such as the larynx, and without lymph node involvement. Thus, the initial workup and isolated nature of disease deemed the patient a good surgical candidate [10–13].

**Fig. 2:**
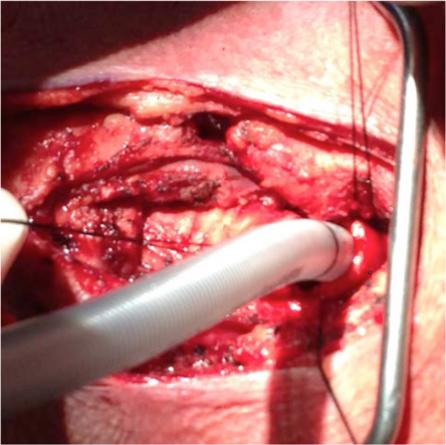
Intraoperative sterile endotracheal tube placed in the trachea following tracheal resection and removal of initial endotracheal tube

**Fig. 3:**
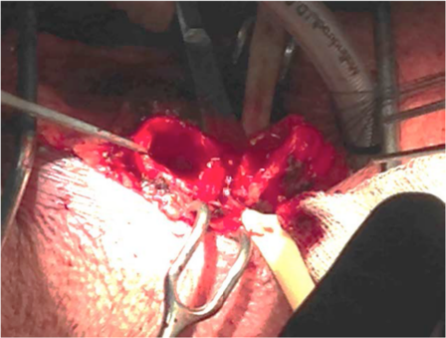
Stay sutures placed in the distal and proximal ends to reapproximate the trachea following tracheal resection

We performed a standard tracheal resection of the tumor located 8 cm from the carina, arising from the posterior membranous trachea, with primary anastomosis (Figs. 2 and 3). Three tracheal rings were resected, including one ring on each side of the mass. The sample was sent off for frozen pathological review, which confirmed negative margins for invasive cancer. An end-to-end reconstruction was then performed. The incision was closed in layers and a Grillo stitch was placed in the patient’s chin to prevent hyperextension in the postoperative period. The patient was extubated immediately postoperatively and taken to the surgical intensive care unit for recovery.

**Fig. 4:**
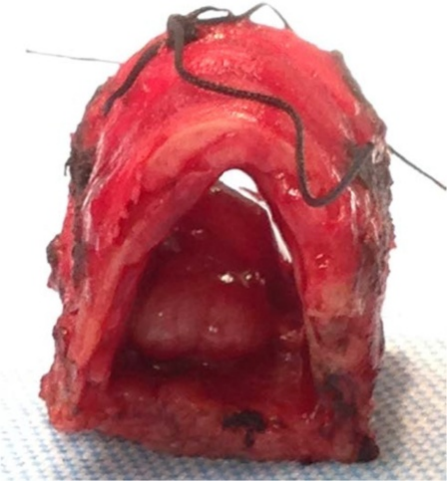
Gross pathology of the sarcomatoid tumor shows a polypoid, firm, tan-white lesion measuring 1.8 cm × 1.5 cm × 0.8 cm, invading 2.5 mm into posterior tracheal wall

**Fig. 5:**
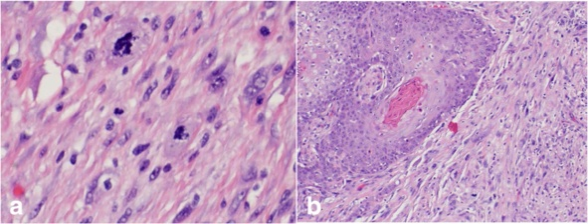
**a** (left): Microscopic features showing enlarged pleomorphic nuclei. **b** (right): Sarcomatoid spindle cell carcinoma with biphasic features of atypical squamous epithelial cells accompanied by enlarged nucleoli and mitosis

Final pathology of the polypoid, firm, tan-white lesion measured 1.8 cm × 1.5 cm × 0.8 cm, invading 2.5 mm into the membranous posterior tracheal wall without involvement of the tracheal cartilage (Fig. 4). Microscopic evaluation was consistent with spindle cell sarcomatoid carcinoma given its biphasic characteristic (Fig. 5). The primary component was a high-grade neoplasm showing enlarged pleomorphic nuclei with spindle cell features. Markedly atypical squamous epithelial cells with enlarged nuclei, prominent nucleoli, and increased mitoses were adjacent to the spindled areas. Along with this thorough pathological review special stains were performed to the specimen to confirm diagnosis in the form of Pankeratin and OSCAR cytokeratin immunostains, which were positive for the atypical squamous cells. No additional molecular testing was performed. Margins were measured at 0.5 cm both proximal and distally with clear posterior radial margin of at least 3 mm All margins were negative for malignancy, and no evidence of lymphovascular or perineural invasion was found. Final pathology correlated with preoperative TNM status as T1aN0M0 as per current classification for tracheal malignancies [8].

Following the procedure, the patient reported improvement in breathing and stridor. After an eight-day hospital course, he self-ambulated on room air, tolerated oral intake, and denied pain or difficulty swallowing or breathing. At his six month post-operative follow-up, he continued to do well without complications or evidence of recurrence. The patient is under observation by both surgery and oncology, with regularly scheduled CT scans to monitor for recurrence.

## Discussion

Surgical management is well established as the definitive treatment for primary tracheal tumors [9, 10, 14]. Resectability of these tumors depends most on locoregional disease characteristics over distant characteristics and is associated with survival superior to palliative therapy, especially when complete resection with negative margins is achieved [12]. The role for adjuvant therapy with chemotherapy or radiation is variable and most recommendations are based on retrospective analysis for non-specific squamous cell histology-associated outcomes [15] and/or for cases where there is advanced disease, other than R0 resection, unresectable tumors or when the patient is not a surgical candidate. Among patients with tracheal tumors, survival in those undergoing complete resections is significantly longer than those undergoing alternative modalities of treatment, although the rarity of these tumors and complexity in selecting treatment options has allowed for limited analysis of outcomes [5, 16].

In the first reported case of primary tracheal spindle cell carcinoma, cisplatin-based chemotherapy with radiation was the treatment chosen by patient preference, however, the regimen was not completed due to patient death within five months of diagnosis [7]. In our case, a complete tracheal sleeve resection was performed, with anastomosis of the regions above and below the resected region (Figs. 2 and 3). Resection alone has thus far proved to be effective in management of this rare case, as the patient has been without complications throughout his postoperative period. Further follow up will be needed to assess the long-term success of this treatment option.

## Conclusion

Spindle cell carcinoma or sarcomatoid carcinoma of the trachea is an uncommon tumor. Because the lesion caused greater than a fifty-percent reduction in trachea luminal diameter, this patient experienced obstructive symptoms such as dyspnea and cough resulting in early detection prior to metastasis. The isolated nature of disease allowed for successful tracheal resection.

As various tracheal tumors have been characterized across reviews, corresponding treatment approaches have widely varied with little evidence pointing towards a single best modality for management [5]. The operative technique described here may serve as a principal reference for future tracheal spindle cell sarcomatoid carcinomas with similar properties. Additionally as more cases are reported, this case contributes to a better understanding of tumor growth predilection along the trachea, tumor size, and possible distant metastasis, allowing for improved assessment of appropriate surgical candidates and higher long-term survival with reduced operative morbidity and mortality.

## Abbreviations

ACC, Adenoid cystic carcinoma; COPD, Chronic obstructive pulmonary disease; CT, Computed tomography; SCC, Squamous cell carcinoma
